# Safety of a Trivalent Inactivated Influenza Vaccine in Health Care Workers in Kurdistan Province, Western Iran; A Longitudinal Follow-up Study

**Published:** 2014-03

**Authors:** Jafar Soltani, Mohamad Jamil Amjadi

**Affiliations:** 1 Department of Pediatrics, Faculty of Medicine, Kurdistan University of Medical Sciences, Sanandaj, Iran;; 2 Sanandaj Center for Disease Control and Prevention, Kurdistan University of Medical Sciences, Sanandaj, Iran

**Keywords:** Influenza vaccine, Adverse reaction, Health care worker, Safety

## Abstract

We studied the safety of a trivalent inactivated surface antigen (split virion, inactivated) influenza vaccine, Begrivac® (Novartis Company), widely used in health care workers in Kurdistan. A longitudinal follow-up study was performed in Sanandaj city, west of Iran, recruiting 936 people. A questionnaire was completed for each participant, and all symptoms or abnormal physical findings were recorded. In part 1 of the study, the post-vaccination complaints were headache (5.3%), fever (7.9%), weakness (9.6%), chills (10.1%), sweating (10.5%), arthralgia (20.2%), and malaise (21.5%). Swelling of the injection site was seen in 267 (30.3%) participants, and pruritus of the injection site was seen in 290 (32.9%) participants. Redness and induration were also reported in 42.5% of the participants. Local reactions were mainly mild and lasted for 1-2 days. No systemic reactions were reported in the second part of the study. None of the participants experienced any inconvenience. We concluded that local adverse reactions after the trivalent inactivated split influenza vaccine, Begrivac®, in health care workers were far more common than expected. Continuous surveillance is needed to assess the potential risks and benefits of newly produced influenza vaccines.

## Introduction

Influenza still remains a global threat. The most effective way to prevent the disease or its severe outcomes is vaccination. Health care workers, especially those who work in hospitals, have frequent contacts with high-risk patients and if they are not vaccinated, they can be the main source of nosocomial transmission of influenza. They may also continue working while ill. It is believed that they can be the sources of many outbreaks in hospitals.^1^

Nosocomial influenza is associated with excess costs because of the long hospital stay, infection control measures, additional tests, and treatment.^2^ It also leads to absence from work and, therefore, interferes with health care providence. An American study done by the Center for Disease Control (CDC) in 221 health care centers revealed that they had a 35% shortage in hospital personnel during the peak season of influenza.^2^ The presence and availability of health care workers are essential in order to provide an efficient response to an influenza pandemic.

For more than 20 years, the CDC has strongly recommended that all health care workers be vaccinated against influenza.^3^ However, the rates of influenza vaccination among health care workers are inappropriately low worldwide, and rarely exceed 40%.^4^^,^^5^ Fear of vaccine side effects is the most common reason for health care workers' reluctance for vaccination. Continuous education regarding the benefits and safety of influenza vaccination can help reach the World Health Organization's (WHO) target of 75% vaccination coverage among health care workers.^6^

We aimed to study the safety of a trivalent inactivated surface antigen (split virion, inactivated) influenza vaccine, Begrivac® (Novartis Company), in health care workers. This vaccine was registered and approved by the Iranian Ministry of Health and Higher Education and used most commonly on the market in Iran. To our knowledge, there have been no studies indicating the adverse reaction of influenza vaccination of health care workers in Iran.

## Materials and Methods

This longitudinal follow-up study was conducted in the Center for Disease Control and Prevention in Sanandaj city, Kurdistan Province, west of Iran. The center is affiliated to Kurdistan University of Medical Sciences. The Research Committee of Kurdistan CDC approved the study. Until the end of 2008, 7000 health care workers were employed by Kurdistan University of Medical Sciences. This study was carried out during the 2008-2009 influenza season (October 2008 through March 2009) and consisted of two parts: an early follow-up visit for 2 weeks and a late follow-up visit for 6 months after vaccination. Healthy health care personnel (HCP) in direct contact with patients were invited to take part. They consisted of physicians, nurses, and other HCPs (including technicians, emergency paramedical service personnel, and laboratory personnel). Allergy to eggs was contraindication for vaccination. Other exclusion criteria were history of severe reaction to previous doses of influenza vaccine including Guillain–Barré syndrome, current severe sickness, underlying chronic diseases, underlying immunodeficiency, or taking immunosuppressive drugs. The vaccine was given free of charge, and it was not obligatory. A total of 936 health care workers were enrolled in the study during this period. This figure is far more than the estimated sample size of 656 calculated by the statistical software. We used the OpenEpi software, Version 2, open source calculator--SSPropor (http://openepi.com/OE2.3/SampleSize/SSPropor.htm). The mean prevalence of adverse events was calculated as 8.2%, withdrawn from the CDC study.^7^ The following rules were used: population size: 7000; anticipated frequency (p): 8.2%; confidence limit: 2%; and design effect: 1.

The antigens for 2007/2008 influenza vaccine were A/Solomon Islands/3/2006 (H1N1)-like strain, A/Wisconsin/67/2005 (H3N2)-like strain, and B/Malaysia/2506/2004-like strain. These antigens complied with the WHO's recommendation (northern hemisphere) and EU's decision for the 2007/2008 season. The vaccine was supplied in pre-filled syringes containing 0.5 ml of vaccine. The trivalent inactivated influenza vaccine has an efficacy of 70-90% in HCP aged 18-64 years when the vaccine and circulating viruses are antigenically matched. The efficacy is lower when these two viruses are not well matched.^1^

Upon vaccination, a CDC staff completed a questionnaire regarding the demographic data of the participants. The process of the study and potential reaction were carefully explained to the participants. By signing at the end of the questionnaire, the HCP had agreed to participate in the study. This questionnaire was used to record any signs and symptoms, including fever or other adverse reactions (local or systemic) observed within a 14-day period after vaccination, regardless of the severity of the symptom. The same questionnaire was used in the following 6 months. In the early follow-up period, all the health care workers were examined weekly by a physician, and all symptoms and abnormal physical findings during the prior days were reviewed and recorded. In the second part of the study, the participants were followed up on a monthly basis by telephone and re-examined upon indication.

Standard definitions for local reactions at or near the injection site were reviewed and used in our study as well as a guideline for case definition.^8^

## Results

Totally, 880 (94%) questionnaires were completed and returned in the first stage of the study and 851 (91%) questionnaires in the second stage of the study. In the first stage of the study, post-vaccination complaints were headache (5.3%), fever (7.9%), weakness (9.6%), chills (10.1%), sweating (10.5%), arthralgia (20.2%), and malaise (21.5%.( All the adverse events were mild. Swelling of the injection site was seen in 267 (30.3%) participants, and pruritus of the injection site was seen in 290 (32.9%) participants. Redness and induration were also reported by 374 (42.5%) health care workers (table 1). No significant systemic reactions were reported in the second part of the study. Eighteen persons reported transient upper respiratory tract symptoms and diarrhea during the second phase of the study, which potentially could not be related to influenza vaccination side effects in this phase. None of the participants experienced any inconvenience in part 1 or 2.

**Table 1:** Frequency of adverse events after influenza vaccination in our study compared with Adverse Events Reported for all Vaccine (VAERS), CDC, USA, 1991–2001

**Adverse events**	**Kurdistan Study**						** CDC^ 1 ^ report (USA^ 2 ^,VAERS^ 3 ^)^7^**
	**Day 1** **(Numbers)**	**Day 2** **(Numbers)**	**Day 3** **(Numbers)**	**Day 4** **(Numbers)**	**Days 5-14** **(Numbers)**	**Total** **Numbers (%)**	
Headache	35	9	3	0	0	47 (5.3%)	5.5%
Fever	49	15	4	1	1	70 (7.9%)	25.8%
Weakness	67	13	3	1	0	84 (9.6%)	-
Chills	52	29	3	3	1	88 (10.1%)	2.6%
Sweating	64	21	3	3	1	92 (10.5%)	1.8%
Arthralgia-Myalgia	94	54	21	6	3	178 (20.2%)	6.4%
Malaise	92	65	21	7	4	189 (21.5%)	3.2%
Redness	161	147	60	3	3	374 (42.5%)	11%
Swelling	114	113	33	7	0	267 (30.3%)	10.8%
Pruritus	96	102	69	10	13	290 (32.9%)	6.9%

^
1 ^CDC: Center for Disease Control; ^2 ^USA: United States of America; ^3 ^VAERS: Vaccine Adverse Event Reporting System

**Figure 1:**
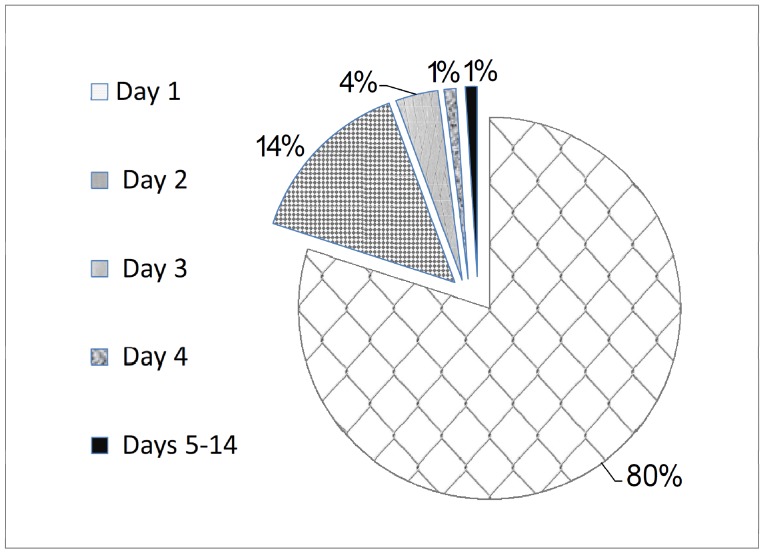
Times of the presentation of the symptoms.

Eighty percent of the adverse events began on the first day of vaccination, 14.4% on the second day, 3.6% on the third day, 1% on the fourth day, and 1% between days 5 to 14 of vaccination (figure 1).

**Figure 2:**
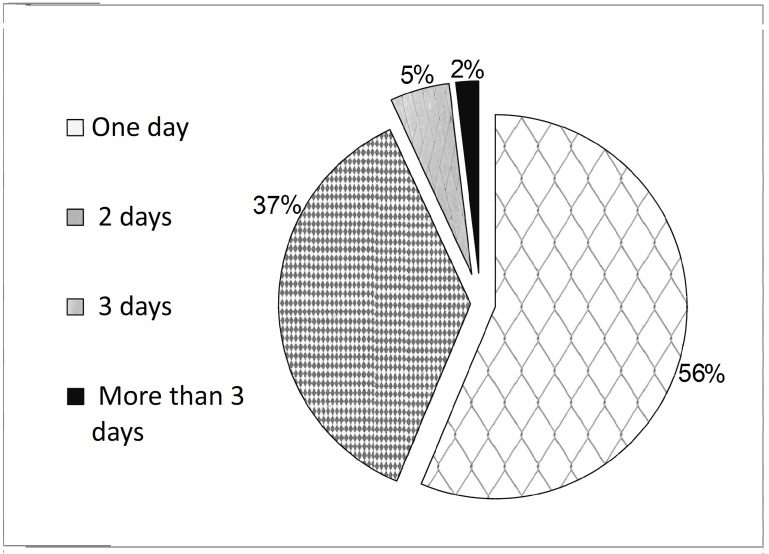
Duration of the symptoms.

Local reactions were mainly mild and lasted for 1 or 2 days. Also, 56.3% of the adverse symptoms lasted for less than 24 hours, 36.8% of the symptoms lasted for less than 2 days, 5% lasted beyond 2 days but less than 3 days, and approximately 1.9% lasted for 4 days. Overall, 98.1% of the health care workers improved within 3 days (figure 2).

## Discussion

In our study, the most frequent local reactions (affecting 30-43% of the participants) were redness, pruritus, and swelling at the vaccination site, typically lasting for less than 2 days. Local reactions were characteristically mild and seldom interfered with the person’s ability to conduct normal daily activities.

Most studies have found a low incidence of local adverse reactions (up to 20%) to influenza vaccination. The results of a report by American Center for Diseases Control indicated that in general, the most common local adverse event was hypersensitivity of the injection site (15.8%), followed by rash (11.0%) and edema of the injection site (10.8%). At least one of these adverse events was present in 74.2% of all the reports by Vaccine Adverse Event Reporting System.^7^ In several studies among adults, the most frequent side effect of vaccination was soreness at the vaccination site (affecting 10-64% of patients).^1^^,^^9^ In our study, local adverse events were encountered more often than expected. Because the health care workers received the questionnaires before vaccination, they were focusing on side effects during the first 48 hours and may have overreported the side effects. Another reason may be that a great proportion of the workers had no history of influenza vaccination and lacked immunity to influenza. Among adults vaccinated in consecutive years, frequencies of adverse effects decreased in the second year of vaccination.^10^

Fever occurred in fewer individuals compared with the cases reported by the American CDC (7.9% vs. 25.8%).^7^ Systemic symptoms, including fever, malaise, and myalgia most often affect people (e.g., infants) with no prior exposure to influenza virus antigens.^11^ Such reactions usually begin 6-12 hours after vaccination and can persist for 1-2 days. In a controlled trial, only body aches (25.1%) were more frequently reported after vaccination with inactivated influenza vaccine compared with placebo injections (20.8%).^12^ Another placebo-controlled trial showed that among healthy adults, administration of split-virus influenza vaccine was associated with significant higher rates of myalgias, arthralgias, fever, and fatigue compared with placebo injections. However the majority of the events were mild.^13^

Immediate allergic reactions such as urticaria and anaphylaxis rarely occur after influenza vaccination.^11^ These reactions mostly result from hypersensitivity to residual egg protein and less likely to thiomersal. The estimated risk of the Guillain-Barré syndrome is reported to be approximately one additional case per million persons vaccinated, with the total number of cases peaking 2 weeks after vaccination.^11^^,^^14^ However, in our study there were no severe adverse reactions such as allergic reactions or the Guillain-Barré syndrome.

Extensive efforts are needed to control influenza. Because health care workers provide care for patients at a high risk for developing complications related to influenza, they should be considered as a priority for expanding influenza vaccine usage. Given the low rates of influenza vaccination among our health care workers (<14%), implementing policies to increase influenza vaccine coverage is critical. A mandatory influenza vaccination policy for health care workers, exempting only those with a medical contraindication, seems to be a highly effective approach for achieving high vaccine coverage among this group of people.^15^ Achieving and sustaining high vaccination coverage among health care workers will protect staff and their patients, and reduce disease burden and health care costs. Educating the staff regarding the minimal side effects of vaccination has a central role in this regard. It should be emphasized that vaccine-related side effects are minimal and have had limited to no impact on the rates of absence from work in health care workers. Education should be accompanied by providing evidence-based documents about the effectiveness and safety of the vaccine.

One of the limitations of our study is that it was based on questionnaires completed by health care workers and, therefore, the answers were subjective. Consequently, personal biases could have influenced the results regarding the rate of adverse reaction and the duration of symptoms.

Our study was disadvantageous because there was no control group and the calculation of relative risk was not possible. Moreover, as there was no randomization, the study sample may not be representative of the population of health care workers. 

## Conclusion

Local adverse reactions after influenza vaccination were far more common than expected. Most of these reactions were mild and transient and did not outweigh the beneficial effects of influenza vaccination in health care workers. The trivalent inactivated split influenza vaccine, Begrivac®, seems to be safe and well tolerated. Continuous surveillance is needed to assess the potential risks and benefits of newly produced influenza vaccines.

## References

[1] Pearson ML, Bridges CB, Harper SA (2006). Influenza vaccination of health-care personnel: recommendations of the Healthcare Infection Control Practices Advisory Committee (HICPAC) and the Advisory Committee on Immunization Practices (ACIP). MMWR Recomm Rep.

[2] Poland GA, Tosh P, Jacobson RM (2005). Requiring influenza vaccination for health care workers: seven truths we must accept. Vaccine.

[3] Atkinson WL, Pickering LK, Schwartz B, Weniger BG, Iskander JK, Watson JC (2002). General recommendations on immunization. Recommendations of the Advisory Committee on Immunization Practices (ACIP) and the American Academy of Family Physicians (AAFP). MMWR Recomm Rep.

[4] Christini AB, Shutt KA, Byers KE (2007). Influenza vaccination rates and motivators among healthcare worker groups. Infect Control Hosp Epidemiol.

[5] Centers for (2010). Interim results: influenza A (H1N1) 2009 monovalent and seasonal influenza vaccination coverage among health-care personnel - United States, August 2009-January 2010. MMWR Morb Mortal Wkly Rep.

[6] Martinello RA, Jones L, Topal JE (2003). Correlation between healthcare workers' knowledge of influenza vaccine and vaccine receipt. Infect Control Hosp Epidemiol.

[7] Zhou W, Pool V, Iskander JK, English-Bullard R, Ball R, Wise RP (2003). Surveillance for safety after immunization: Vaccine Adverse Event Reporting System (VAERS)--United States, 1991-2001. MMWR Surveill Summ.

[8] Gidudu J, Kohl KS, Halperin S, Hammer SJ, Heath PT, Hennig R (2008). A local reaction at or near injection site: case definition and guidelines for collection, analysis, and presentation of immunization safety data. Vaccine.

[9] Ahmed F, Singleton JA, Franks AL (2001). Clinical practice. Influenza vaccination for healthy young adults. N Engl J Med.

[10] Ohmit SE, Gross J, Victor JC, Monto AS (2009). Reduced reaction frequencies with repeated inactivated or live-attenuated influenza vaccination. Vaccine.

[11] Fiore AE, Uyeki TM, Broder K, Finelli L, Euler GL, Singleton JA (2010). Prevention and control of influenza with vaccines: recommendations of the Advisory Committee on Immunization Practices (ACIP), 2010. MMWR Recomm Rep.

[12] Neuzil KM (2002). The safety of inactivated influenza vaccine adults and children with asthma. J Pediatr.

[13] Jackson LA, Gaglani MJ, Keyserling HL, Balser J, Bouveret N, Fries L (2010). Safety, efficacy, and immunogenicity of an inactivated influenza vaccine in healthy adults: a randomized, placebo-controlled trial over two influenza seasons. BMC Infect Dis.

[14] Haber P, DeStefano F, Angulo FJ, Iskander J, Shadomy SV, Weintraub E (2004). Guillain-Barré syndrome following influenza vaccination. JAMA.

[15] Pavia AT (2010). Mandate to protect patients from health care-associated influenza. Clin Infect Dis.

